# Non-surgical periodontal therapy and systemic inflammatory biomarkers in patients with periodontitis and carotid plaque burden: a pilot study

**DOI:** 10.3389/fcimb.2026.1881815

**Published:** 2026-08-21

**Authors:** Ana Maria Hofer, Andrei Picos, Alexandra Dădârlat-Pop, Raluca Tomoaia, Horia Rosianu, Ilyés Tamás, Monica Popa

**Affiliations:** 1 Department of Community Health, “Iuliu Hatieganu” University of Medicine and Pharmacy, Cluj-Napoca, Romania; 2 Department of Oral Prevention, “Iuliu Hatieganu” University of Medicine and Pharmacy, Cluj-Napoca, Romania; 3 Department of Cardiology, Heart Institute “N. Stăncioiu”, Cluj-Napoca, Romania; 4 4^th^ Department of Internal Medicine, Faculty of Medicine, “Iuliu Hatieganu” University of Medicine and Pharmacy, Cluj-Napoca, Romania; 5 Department of Cardiology, Rehabilitation Hospital, Cluj-Napoca, Romania; 6 Department of Molecular Sciences, “Iuliu Hatieganu” University of Medicine and Pharmacy, Cluj-Napoca, Romania

**Keywords:** cardiovascular risk, carotid plaque burden, inflammatory biomarkers, periodontitis, systemic inflammation

## Abstract

**Introduction:**

Systemic inflammation is a key contributor to the pathophysiology of carotid plaque burden (CpB). Increasing evidence supports a link between periodontitis and systemic conditions, including endothelial dysfunction, and CpB. This study aimed to explore inflammatory mediators involved in cardiovascular disease that may represent shared mechanistic pathways between periodontitis and carotid plaque burden, and to determine whether non-surgical periodontal therapy modulates systemic inflammatory activity.

**Methods:**

A pilot study was conducted on subjects presenting with both periodontitis and CpB. Of 87 initially screened participants, 10 met the inclusion criteria and completed the study. Periodontal parameters—probing pocket depth (PPD), clinical attachment loss (CAL), and bleeding on probing (BOP)—were recorded. Systemic inflammatory biomarkers, including matrix metalloproteinase-8 (MMP-8), myeloperoxidase (MPO), lipoprotein-associated phospholipase A2 (Lp-PLA2), and soluble CD40 ligand (sCD40L), were analyzed.

**Results:**

Participants demonstrated severe periodontal disease, with mean PPD of 5.5 mm (maximum 8 mm), mean CAL of 6.46 mm (maximum 12 mm), and BOP of 67%. Periodontal therapy was associated with significant reductions for MMP-8 (25.64 ± 20.31 vs. 10.60 ± 3.41ng/mL post-treatment; p = 0.010) and Lp-PLA2 (p = 0.0195). MPO and sCD40L showed decreasing trends that did not reach statistical significance (p = 0.097 and p = 0.156, respectively). Correlation analyses demonstrated limited associations between baseline biomarker levels and periodontal clinical parameters, except for an inverse correlation between sCD40L and probing pocket depth (ρ = −0.77, p = 0.015).

**Conclusion:**

Inflammatory biomarkers may represent important mechanistic links between periodontitis and imaging-confirmed carotid atherosclerotic plaques. Within the limitations of this pilot study, non-surgical periodontal therapy was associated with reductions in selected systemic inflammatory biomarkers, supporting the feasibility of investigating the periodontitis–carotid plaque axis in larger translational cohorts. Larger studies are needed to validate these findings.

## 1 Introduction

Carotid plaque burden is a well-recognized risk factor for ischemic stroke, accounting for approximately 10–20% of strokes and transient ischemic attacks (Brinjikji et al., 2016). The visualization and quantification of subclinical atherosclerosis using noninvasive vascular imaging are playing an increasingly important role in cardiovascular risk assessment. There are studies showing that subclinical atherosclerosis burden, including carotid plaque burden (CpB) in asymptomatic individuals was independently associated with all-cause mortality, and disease progression was likewise independently predictive of mortality risk (Fuster et al., 2024). So, atherosclerosis is a chronic, degenerative disorder affecting large- and medium-sized arteries. It is characterized anatomically by the formation of lipid-rich, fibrocellular lesions—composed of viable cells and extracellular matrix (ECM) components—that disrupt the normal histological architecture of the intima and encroach upon the vascular lumen. These lesions are commonly known as atheromas or atherosclerotic plaques (Bentzon et al., 2014). Even though, the atherosclerotic process begins early in life; however, it is influenced by a complex interplay of intrinsic and extrinsic factors that modulate its initiation and progression. Thrombosis secondary to plaque rupture or superficial erosion represents a major complication of atherosclerosis and may lead to sudden luminal occlusion, resulting in acute ischemic syndromes. Infectious agents may also contribute to the inflammatory response, thereby promoting lesion destabilization (De Meyer et al., 2024). Systemic inflammation plays a significant role in the pathophysiology of CpB and can be evaluated using several inflammatory biomarkers, which may be linked to a higher risk of complications and increased mortality in patients with CpB (Scalise et al., 2025). CpB was selected as the vascular parameter of interest because imaging-confirmed carotid plaques represent a clinically relevant manifestation of subclinical carotid atherosclerosis.

On the other hand, periodontitis is a complex, multifactorial inflammatory condition that arises in response to periodontal pathogens, leading to the progressive destruction of the supporting structures of the teeth. If left untreated, this process can ultimately result in tooth loss (Tonetti et al., 2018). Increasing evidence from recent studies has demonstrated a significant association between periodontitis and various systemic disorders, including diabetes mellitus, endothelial dysfunction, and carotid artery stenosis (CS) (Ismail et al., 2023; De Meyer et al., 2024; Fuster et al., 2024; Widen et al. 2020; Polizzi et al., 2020).

Carotid atherosclerotic plaques develop because of progressive lipid accumulation and chronic inflammation within the carotid arterial wall and may eventually lead to carotid stenosis and ischemic cerebrovascular events. Clinical manifestations range from transient visual disturbances and neurological deficits to ischemic stroke, although the disease often remains asymptomatic until advanced stages, highlighting the importance of early detection and risk factor management.

The pathogenesis of carotid atherosclerosis parallels that of atherosclerosis in general, beginning with endothelial dysfunction and progressing to the formation of a fibrous cap overlying a lipid-rich necrotic core containing foam cells. Increasing evidence suggests that several inflammatory mediators contribute to the pathophysiology of both periodontitis and carotid atherosclerotic disease. These include the secretion and release of toxic metabolites (Bilgin Ç etin et al., 2020; Holmlund et al., 2010; Schmidt et al., 2018), along with proinflammatory cytokines such as interleukins (ILs) and matrix metalloproteinases (MMPs) (Li et al., 2014; Senini et al., 2019).

Periodontitis and CpB have been shown to share common etiopathogenetic mechanisms. Several studies have sought to establish correlations between the presence of oral bacteria within atherothrombotic tissues and their detection in subgingival plaque or serum samples from the same individuals. These investigations suggest that patients with periodontitis exhibit an increased risk of CS (Schenkein and Loos, 2013; Sanz et al., 2020). The proposed pathophysiological mechanisms underlying this association involve transient bacteremia and subsequent systemic inflammatory responses, characterized by elevated levels of C-reactive protein and enhanced oxidative stress (Schenkein and Loos, 2013).

Evidence indicates that both periodontitis and CpB are characterized by a marked increase, at local (oral) and systemic levels, in a range of inflammatory mediators: C-reactive protein (CRP), prostaglandins, interleukins (IL-1, IL-6, IL-10), and various matrix metalloproteinases (MMPs), which are elevated in response to host immune activation triggered by specific periodontal pathogens (Schenkein and Loos, 2013; Noack et al., 2017; Lahdentausta et al., 2019; Sanz et al., 2020). Notably, several studies have demonstrated that MMP expression is significantly upregulated in periodontitis compared to gingivitis or periodontal health, suggesting that dysregulated MMP activity contributes to the accelerated destruction of periodontal tissues (Bastos et al., 2016; Widén et al., 2016). Moreover, among matrix metalloproteinases, MMP-9 has been widely recognized as a key mediator of inflammatory progression in both periodontitis (Noack et al., 2017) and CpB (Yabluchanskiy et al., 2013; Rupprecht et al., 2020). It is primarily released by activated macrophages in response to pathogenic stimuli and serves as a subclinical marker of vascular homeostasis (Nagase, 1997). MMP-9 modulates several proinflammatory mediators, including IL-1, IL-6, IL-8, and prostaglandins (Boelen et al., 2019; Kim et al., 2020), and its elevated expression has been correlated with active periodontal tissue destruction (Chang et al., 2004; Kim et al., 2020). Increased MMP-9 levels in gingival crevicular fluid (GCF) have been linked to early periodontitis (Schenkein and Loos, 2013; Ionel et al., 2017), suggesting a role in neoangiogenesis during host–pathogen interactions (Holtfreter et al., 2013). Furthermore, MMP-9, in concert with C-reactive protein (CRP), may suppress nitric oxide (NO) bioavailability, potentially impairing endothelial function and increasing CVD risk (Gurav, 2014). Matrix metalloproteinase-8 (MMP-8), together with myeloperoxidase (MPO), lipoprotein-associated phospholipase A2 (Lp-PLA2), and soluble CD40 ligand (sCD40L)—play distinct but complementary roles in the pathophysiology of atherosclerosis and plaque instability (Herman et al., 2001; Rahman et al., 2013; Li et al., 2026). MMP-8, a collagen-degrading enzyme, contributes to extracellular matrix breakdown and weakening of the fibrous cap, thereby promoting plaque vulnerability (Herman et al., 2001). MPO, released by activated neutrophils and monocytes, generates reactive oxygen species and enhances oxidative stress, leading to endothelial dysfunction and lipid oxidation (Herman et al., 2001).

Lp-PLA2, primarily associated with low-density lipoproteins, hydrolyzes oxidized phospholipids and produces pro-inflammatory mediators that further drive vascular inflammation and plaque progression (Li et al., 2026).

sCD40L, expressed by activated platelets and immune cells, mediates inflammatory signaling and promotes thrombogenic activity, linking inflammation with thrombus formation. There is evidence that indicates that elevated levels of sCD40L are independently associated with increased cardiovascular risk in patients with asymptomatic low-grade CS (Lin et al., 2024).

Together, these markers reflect key mechanisms underlying atherosclerotic disease, including inflammation, oxidative stress, matrix degradation, and thrombosis, making them valuable indicators of cardiovascular risk and plaque instability. These findings underscore the growing interest in identifying early biomarkers to assess subclinical susceptibility to both periodontitis and CVD. Advances in understanding the pathophysiology of CpB are opening new avenues for prevention and treatment. In particular, recognition of the central role of inflammation has highlighted the potential for therapeutic strategies aimed at selectively inhibiting the inflammatory cascade within the vessel wall. Targeting inflammatory triggers may contribute to improved clinical outcomes in patients with CpB.

Therefore, based on this evidence, this study aimed to evaluate the serum levels of MMP-8, MPO, Lp-PLA2, sCD40L in patients with periodontitis and CpB. The primary aim of this pilot study was to explore whether non-surgical periodontal therapy is associated with short-term changes in circulating inflammatory mediators implicated in both periodontitis and carotid plaque burden. A secondary aim was to examine the direction and strength of associations between baseline biomarker levels and periodontal clinical parameters.

## 2 Materials and methods

This study was designed as an exploratory pilot investigation intended to assess feasibility and preliminary biomarker trends in patients with concomitant periodontitis and carotid plaque burden. Therefore, no formal *a priori* sample size calculation was performed. The number of screened participants reflected the recruitment capacity during the predefined study period at the participating center, “Niculae Stăncioiu” Heart Institute in Cluj-Napoca.

To ensure a homogeneous sample and minimize potential confounding factors related to age and sex, the participants were aged 33–76 years. Data were collected for all participants, including demographic characteristics such as sex, age, and educational level. Clinical and lifestyle-related variables were also recorded, including body mass index (BMI), medication use, and the presence of systemic comorbidities.

The overall health status was assessed using complementary imaging investigations, including carotid Doppler ultrasonography or carotid CT angiography, followed by venous blood sampling for laboratory analyses, all performed at the “Niculae Stăncioiu” Heart Institute in Cluj-Napoca. The study group included patients who underwent ultrasonographic evaluation of the supra-aortic trunks at the Heart Institute during September-November 2024.

Subsequently, participants underwent a periodontal examination at the Picos Dental Clinic (16 Avram Iancu Street, Cluj-Napoca). Periodontal parameters were recorded at six sites per tooth, and two indices — the Oral Hygiene Index and the Bleeding Index — were calculated. These procedures were non-invasive and minimally invasive.

The periodontal treatment consisted of three phases: non-surgical periodontal therapy, involving subgingival instrumentation; carious lesions treatment and infection control; re-evaluation, during which all periodontal indices and measurements were reassessed. At three months post-treatment, a follow-up examination was performed, and venous blood samples were collected again to determine the concentrations of proinflammatory mediators.

Non-surgical periodontal therapy was completed in two to four sessions, depending on disease severity and patient compliance. Subgingival instrumentation was performed using ultrasonic scalers and Gracey curettes until complete removal of supra- and subgingival deposits was achieved. No systemic antibiotics or adjunctive local antimicrobial agents were prescribed during the study period. Patients were instructed to rinse twice daily with 0.12% chlorhexidine for 14 days following instrumentation. All participants received individualized oral hygiene instructions, including toothbrushing technique and interdental cleaning recommendations. Reevaluation was performed 3 months (± 2 weeks) after completion of active periodontal therapy.

### 2.1 Inclusion and exclusion criteria

For patients with periodontitis (Tonetti et al., 2018), inclusion criteria were:

1. Presence of ≥16 natural teeth.
2. Interdental clinical attachment loss (CAL) of ≥ 2 non-adjacent teeth.
3. Buccal or oral CAL ≥ 3 mm with pocketing ≥ 3 mm, detectable at ≥ 2 teeth.

At least one site exhibiting ≥2 mm crestal bone loss confirmed radiographically using periapical technique (Tonetti et al., 2018).

Among patients with CpB, eligibility required an age of ≥18 years, no prior history of cardiovascular disease (myocardial infarction, stroke, angina, heart failure, or arterial revascularization), no ongoing cancer treatment, and no conditions limiting long-term participation.

Exclusion criteria included: (1) severe allergies; (2) antibiotic, anti-inflammatory, or immunosuppressive therapy within 3 months prior to enrollment; (3) alcohol consumption; (4) use of contraceptives; (5) pregnancy or lactation; (6) medications inducing gingival hypertrophy or hyperplasia; (7) periodontal treatment within 3 months before the study.

The exclusion criteria were applied to reduce potential confounding factors that might independently influence systemic inflammatory biomarker levels and thereby affect the interpretation of treatment-related changes.

### 2.2 Carotid plaque burden assessment

CpB was assessed at baseline using a high-resolution, 2-dimensional 9L-D linear array transducer using a Vivid E95 ultrasound system (GE HealthCare), with scanning in longitudinal and cross-sectional orientations from the proximal common carotid artery to the distal internal carotid artery on each side. On carotid ultrasound, an atherosclerotic plaque was defined as a focal thickening of the vessel wall, typically measuring > 1.5 mm from the intima-lumen interface to the media-adventitia interface, or a thickening that was 50% greater than the surrounding intima-media thickness (IMT). Plaques were classified by their ultrasound appearance—soft/hypoechoic (fat-rich, unstable) or hard/hyperechoic (calcified, stable). In the present study, carotid plaque burden was operationally defined as the presence of imaging-confirmed carotid atherosclerotic plaques rather than as a quantitative plaque burden score. Parameters such as plaque number, maximal plaque thickness, plaque area, plaque volume, or total plaque score were not systematically recorded.

At the initial visit, medical and dental data were obtained, including cardiovascular diagnostic evaluations (e.g., electrocardiography and carotid ultrasound), as well as venous blood samples. Individuals with both periodontitis and CpB fulfilled the inclusion criteria for both groups.

**Figure 1:**
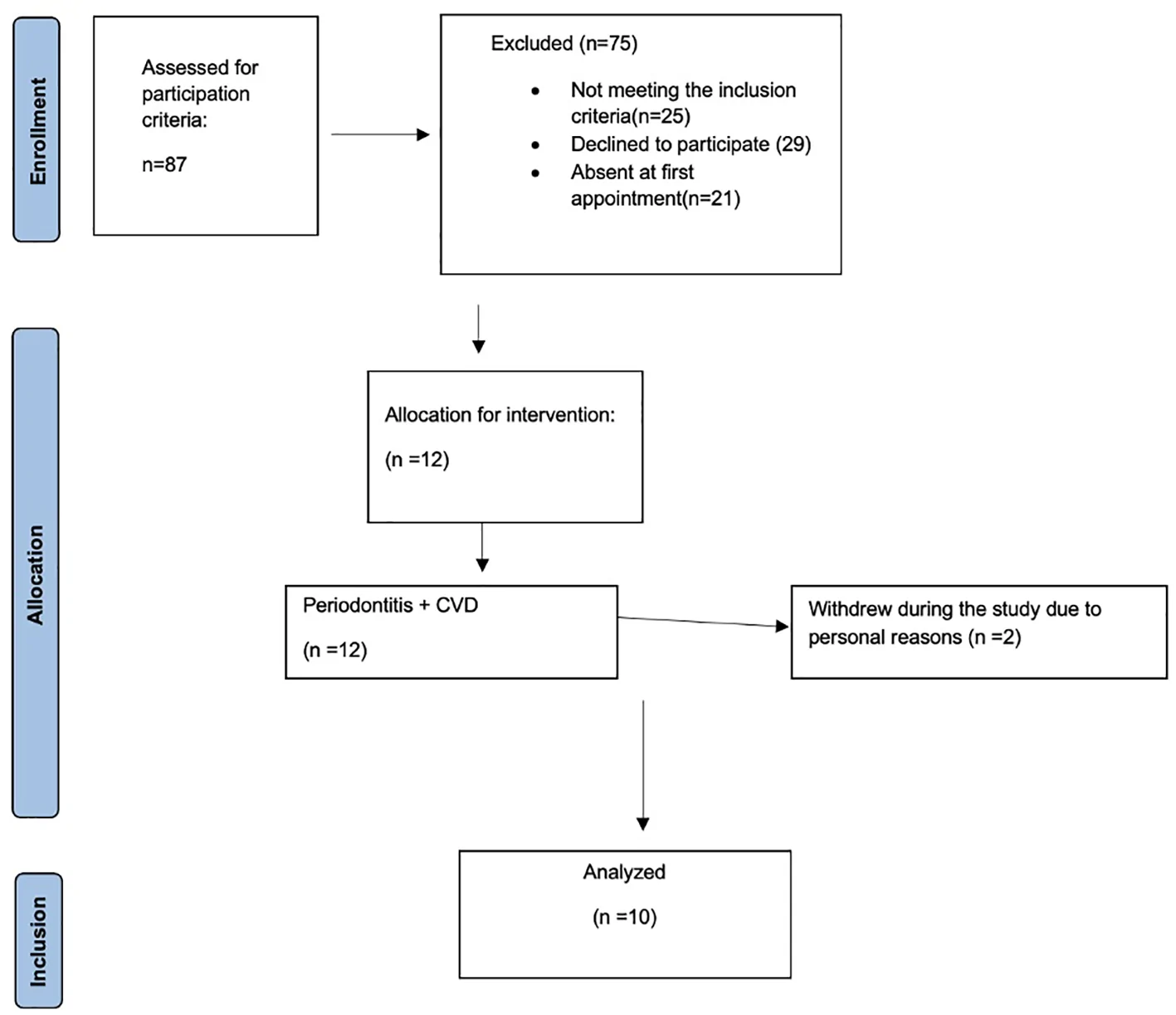
Flow diagram illustrating participant selection, allocation, and analysis in the study.

Of 87 initially screened participants, 25 were excluded for not meeting inclusion criteria, 29 declined participation, 21 did not attend the baseline visit and 2 did not attend the reevaluation visit. Ultimately, 10 subjects were included with both periodontitis and CpB – **Figure 1**.

### 2.3 Laboratory analyses

Fasting antecubital venous blood samples were collected in the morning prior to the procedure. Serum and plasma were separated by centrifugation at 3.000g for 10 minutes and stored at −80 °C until analysis. The concentrations of MMP-8, MPO, Lp-PLA2, and sCD40L were determined using commercially available enzyme-linked immunosorbent assay (ELISA) kits, according to the manufacturers’ instructions. The lower limits of detection were as follows: MMP-8, 0.10 ng/mL; MPO, 0.028 ng/mL; Lp-PLA2, 0.19 ng/mL; and sCD40L, 0.0375 ng/mL. All measurements were performed in duplicate, and the mean values were used for statistical analysis. Intra- and interassay coefficients of variation were all under 10%.

### 2.4 Assessment of traditional cardiovascular risk factors

In addition, major cardiovascular risk factors were systematically assessed – **Table 1**. Cardiovascular risk factors were assessed through a combination of patient interviews, medical record review, physical examination, and laboratory measurements. Hypertension was defined as a history of antihypertensive treatment or repeated blood pressure measurements ≥140/90 mmHg. Diabetes mellitus was defined as a prior diagnosis, use of antidiabetic medication, or fasting plasma glucose ≥126 mg/dL. Dyslipidemia was defined as total cholesterol ≥200 mg/dL, low-density lipoprotein cholesterol (LDL-C) ≥130 mg/dL, triglycerides ≥150 mg/dL, high-density lipoprotein cholesterol (HDL-C) <40 mg/dL in men or <50 mg/dL in women, or current lipid-lowering therapy. Smoking status was categorized as current, former, or never smoker. Obesity was defined as a body mass index (BMI) ≥30 kg/m², according to standard guidelines.

**Table 1:** Cardiovascular risk factors and parameters that provide an overview of individual cardiovascular risk profiles.

No.	Age	Sex	Total cholesterol (mg/dL)	HDL	LDL	Triglycerides	CRP	ESR	Weight (kg)	Height (cm)	BMI (kg/m²)	Diabetes mellitus	Smoking
1	52	F	152	43.3	93	138	12.4	7	82	161	31.63	no	no
2	71	M	228	40.2	160	210	11.3	6	90	180	27.8	no	no
3	76	M	120	38.9	182	270	16.5	10	95	182	28.7	no	no
4	67	M	215	35.7	162	267	9.7	15	89	174	29.4	no	no
5	67	M	233	60.1	176	211	6.9	14	97	175	31.7	no	no
6	57	M	260	58.2	188	167	12.5	14	93	169	32.6	yes	no
7	33	M	254	62.1	132	180	13.5	18	125	180	38.6	no	no
8	59	M	284	36.6	87.9	220	7.6	16	88	180	26.5	no	no
9	44	M	241	66.2	102	150	38.7	45	84	174	27.7	yes	no
10	52	M	205	66.2	98	156	13.8	17	82	176	26.5	yes	yes

Comprehensive periodontal charting was performed for each patient, including measurements of clinical attachment loss (CAL), probing depth (PD), bleeding on probing (BOP), and plaque index (PI) (Li et al., 2026). CAL was determined using the cementoenamel junction as a reference point and calculated as the sum of PD and gingival recession. All periodontal parameters were recorded at six sites per tooth for all teeth present in the dental arch. All parameters were recorded by two independent calibrated examiners (principal and control examiner) using a periodontal probe (UNC-15, Hu-Friedy, Chicago, IL, USA). The gingival bleeding index (GBI) was calculated as the percentage of sites showing bleeding upon gentle probing out of the total number of sites examined.

### 2.5 Statistical analysis

All statistical analysis was carried out using RStudio Desktop (RStudio^©^, PBC RStudio v2026.01.0 + 392, Boston, MA, USA). Prior to comparative analysis, the distribution of quantitative variables was assessed using the Shapiro-Wilk test for normality.

For variables that followed a normal distribution, paired Student’s t-test was applied to compare pre- and post-intervention values. When the data did not follow a normal distribution, the Wilcoxon signed-rank test was used instead of the paired Student’s t-test. The Spearman correlation coefficient and its corresponding p-value were used to assess the correlation between non-normally distributed quantitative variables. For all statistical tests, the two-tailed p-value was reported, and a 0.05 value was considered as the level of statistical significance. Serum concentrations of MMP-8, MPO and Lp-PLA2 were expressed as ng/mL, whereas sCD40L concentrations were expressed as pg/mL. Periodontal parameters, such as periodontal pocket probing (PPD) and clinical attachment loss (CAL) were expressed in millimeters(mm) and bleeding on probing (BOP) in percentage (%).

All qualitative variables were excluded from the analysis prior to testing.

## 3 Results

All patients enrolled in this study were diagnosed with periodontitis, categorized into stage II as well as stage III/IV, indicating the absence of any other periodontal conditions, with the objective of characterizing both clinical periodontal parameters and inflammatory biomarkers. Continuous variables are reported using mean values, standard deviations, and minimum–maximum ranges.

Clinical periodontal assessment confirmed the presence of moderate to severe periodontal disease within the study group: Probing Depth (PPD) demonstrated a mean value of approximately 5.5 mm, with maximum values reaching 8 mm, consistent with pathological periodontal pocket formation; Clinical Attachment Loss (CAL) showed a mean of 6.46 mm, with values extending up to 12 mm, indicating substantial loss of periodontal support structures; Bleeding on Probing (BOP) presented a mean value of 0.67 (67%), reflecting widespread gingival inflammation and active disease in the majority of patients. **Table 2** summarizes the clinical periodontal characteristics of the study population. Mean probing pocket depth and clinical attachment loss values indicate moderate to severe periodontal destruction. The high mean percentage of bleeding on probing reflects persistent gingival inflammation in most patients.

**Table 2:** Descriptive statistics of clinical baseline periodontal parameters.

Variable	Mean	SD	Minimum	Maximum
PPD (mm)	5.54	2.03	3	8
CAL (mm)	6.46	3.67	2	12
BOP (%)	66.92	21.69	38	98

**Table 2** summarizes the clinical periodontal characteristics of the study population. Mean probing pocket depth and clinical attachment loss values indicate moderate to severe periodontal destruction. The high mean percentage of bleeding on probing reflects persistent gingival inflammation in most patients at the baseline.

Following non-surgical periodontal therapy, patients demonstrated marked clinical periodontal improvement at the 3-month reevaluation. This was characterized by lower mean PPD (3.50 ± 0.53 mm) and BOP (12.00 ± 6.07%), indicating effective resolution of periodontal inflammation. Mean CAL remained unchanged (6.46 ± 3.67 mm), as no measurable attachment gain was expected within the study follow-up period.

Baseline biomarker analysis revealed considerable interindividual variability, reflecting differences in inflammatory activity among participants. Mean baseline MMP-8 concentrations were 25.64 ± 20.31 ng/mL, while MPO, Lp-PLA2, and sCD40L baseline values were reported as median concentrations of 45.5 ng/mL, 46.8 ng/mL, and 9538 pg/mL, respectively. Biomarker analyses were performed on all available samples, and the number of observations included in each analysis is reported in the corresponding tables.

As shown in **Table 3**, inflammatory biomarkers exhibited wide ranges and elevated mean values, indicating marked interindividual variability. Increased MMP-8 and MPO levels may suggest active collagen degradation and neutrophil-mediated inflammation, respectively. Lp-PLA2 and sCD40L values may support the presence of a systemic inflammatory response in patients with periodontitis and CpB.

**Table 3:** Descriptive statistics of inflammatory biomarkers in patients with periodontitis.

Variable	Value	Range
MMP-8 (ng/mL)	25.64 ± 20.31	3.00–78.00
MPO (ng/mL)	45.50 (median)	6.00–197.00
Lp-PLA2 (ng/mL)	46.80 (median)	21.4–167.200
sCD40L (pg/mL)	9538.5 (median)	3524.6–18015.6

**Figure 2:**
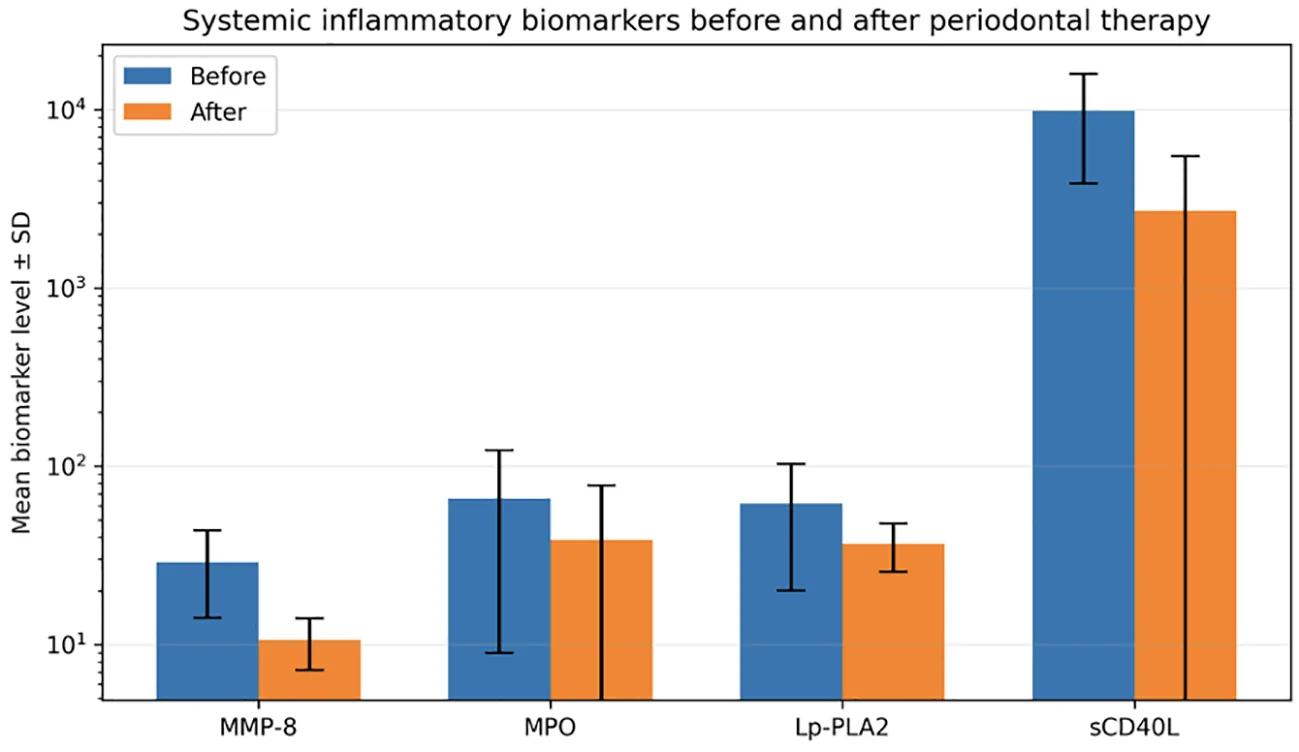
Systemic inflammatory biomarker concentrations before and after periodontal therapy. MMP-8, MPO and Lp-PLA2 concentrations are expressed as ng/mL, while sCD40L concentrations are expressed as pg/mL. Values are presented as mean ± SD for MMP-8 and median values for MPO, Lp-PLA2 and sCD40L.

**Figure 2**. Systemic inflammatory biomarker levels before and after periodontal therapy are presented as mean ± standard deviation.

The results of the paired statistical analysis comparing inflammatory biomarker levels before and after the intervention are presented, using either the paired Student’s t-test or the Wilcoxon signed-rank test, depending on the data distribution.

As shown in **Table 4**, a statistically significant decrease was observed for MMP-8 levels after the intervention (p = 0.010). The mean value decreased from 25.64 ± 20.31 ng/mL at baseline to 10.60± 3.41 ng/mL after treatment. This finding suggests that the intervention was associated with reducing periodontal tissue-destructive processes mediated by MMP-8.

**Table 4:** Reduction in biomarker levels after periodontal therapy, with significant decreases observed for MMP-8 and Lp-PLA2, while MPO and sCD40L exhibited non-significant changes.

Biomarker	Baseline	Post-treatment	P-value
MMP-8 (ng/mL)	25.64 ± 20.31	10.60 ± 3.41	0.010
MPO (ng/mL)	45.50 (median)	23.50 (median)	0.097
Lp-PLA2 (ng/mL)	46.80 (median)	35.30 (median)	0.0195
sCD40L(pg/mL)	9538 (median)	1642 (median)	0.156

Similarly, Lp-PLA2 levels showed a statistically significant reduction following treatment (p = 0.0195), with values decreasing from 46.8 to 35.3. This finding may reflect changes in circulating inflammatory mediator levels and could suggest modulation of inflammatory pathways shared between periodontitis and atherosclerotic disease.

In contrast, although MPO levels decreased numerically from 45.5 to 23.5, this change did not reach statistical significance (p = 0.097). Likewise, sCD40L levels showed a reduction from 9538 to 1642 pg/mL, but the difference was not statistically significant (p = 0.156). These findings suggest a trend toward reduced inflammatory and platelet activation activity; however, the observed changes were insufficient to demonstrate a statistically significant effect within the studied sample size.

In summary, **Table 4** demonstrates a statistically significant post-treatment reduction in MMP-8 and Lp-PLA2 levels, while MPO and sCD40L showed non-significant decreasing trends.

Spearman correlation analysis was performed to evaluate the relationship between baseline circulating inflammatory biomarkers and clinical periodontal parameters. Overall, limited associations were observed between systemic inflammatory marker concentrations and measures of periodontal disease severity.

Baseline MMP-8 levels showed no significant correlations with probing pocket depth, clinical attachment loss or bleeding on probing. Similarly, circulating MPO concentrations were not significantly associated with periodontal parameters, although a moderate positive trend was noted between MPO and clinical attachment loss.

Baseline Lp-PLA2 concentrations also failed to demonstrate significant relationships with periodontal clinical parameters, suggesting that systemic vascular inflammatory activity reflected by this biomarker does not directly parallel local periodontal tissue destruction.

In contrast, a significant inverse correlation was identified between baseline sCD40L levels and probing pocket depth, indicating that higher circulating sCD40L concentrations were associated with lower periodontal pocket depth values. A negative trend was also observed between sCD40L and bleeding on probing, although this did not reach statistical significance.

These findings suggest that most systemic inflammatory biomarkers evaluated in this study were not strongly related to the clinical severity of periodontal disease at baseline (**Table 5**). However, a strong negative correlation was observed between sCD40L and probing pocket depth (PPD) (ρ = −0.77, p = 0.015), indicating that higher sCD40L levels were associated with lower PPD values. Additionally, sCD40L showed a strong negative, although not statistically significant, correlation with bleeding on probing (BOP) (ρ = −0.61, p = 0.081). No other significant correlations were identified.

**Table 5:** Values represent Spearman correlation coefficients (ρ) with corresponding p-values; statistical significance was set at p < 0.05.

Biomarker	PPD	CAL	BOP
MMP-8	−0.08 (0.825)	−0.14 (0.696)	−0.29 (0.413)
MPO	−0.15 (0.650)	0.39 (0.205)	−0.27 (0.402)
Lp-PLA2	0.06 (0.841)	0.13 (0.676)	−0.20 (0.504)
sCD40L	−0.77 (0.015)	0.09 (0.828)	−0.61 (0.081)

## 4 Discussion

Emerging evidence highlights a significant link between periodontitis and an in-creased risk of cardiovascular events, including carotid atherosclerosis (Li et al., 2026). Experimental studies further support this association; in atherosclerotic mouse models, periodontal inflammation is more severe and accompanied by distinct alterations in lipid profiles, suggesting a mechanistic interplay between systemic lipid metabolism and periodontal pathology (Li et al., 2026).

The present exploratory pilot study evaluated systemic inflammatory mediators in patients presenting with concomitant periodontitis and imaging-confirmed carotid plaque burden. The principal findings were: (1) periodontal treatment was associated with significant post-treatment reductions in MMP-8 and Lp-PLA2 levels following non-surgical periodontal therapy; (2) non-significant decreasing trends for MPO and sCD40L; and (3) limited correlations between circulating biomarkers and periodontal clinical parameters.

Despite these findings, there remains a substantial unmet need for effective strategies for early detection and risk stratification in this high-risk population. In this context, the identification of shared inflammatory pathways may provide valuable insights into the biological mechanisms linking periodontal disease and CpB. Therefore, the present study investigated the potential inflammatory links between periodontal disease and CpB in patients without a history of major cardiovascular disease. Previous research has established associations between oral disease and coronary heart disease, coronary events, and stroke; however, the present investigation appears to be among the first specifically designed to characterize multiple inflammatory pathways connecting periodontal disease with subclinical carotid atherosclerosis. Furthermore, to the best of our knowledge, no studies to date have examined the role of the biomarkers investigated in this study in the pathophysiological link between periodontitis and carotid plaque burden.

Carotid plaque burden (CpB) is an important marker of atherosclerotic disease and cardiovascular risk, with plaque progression and vulnerability being closely associated with inflammatory processes (Novo et al., 2005). Several circulating biomarkers, including Lp-PLA2, sCD40L, MMPs, hsCRP, IL-6, and osteopontin, have been implicated in plaque progression, destabilization, and an increased risk of cerebrovascular events (Tonetti et al., 2018; Zhang et al., 2026). Furthermore, advanced imaging modalities such as ultrasound, MRI, and PET enable the assessment of plaque morphology and inflammatory activity, supporting the relationship between systemic inflammation and carotid plaque vulnerability (Ding et al., 2008; Takasaki et al., 2011; Brinjikji et al., 2016; Wijeratne et al., 2020). These observations provide the rationale for investigating whether non-surgical periodontal therapy may influence systemic inflammatory biomarkers in patients with periodontitis and CpB.

Periodontitis and carotid atherosclerosis are highly prevalent conditions, and accumulating evidence suggests that periodontitis may act as a non-traditional cardiovascular risk factor (Beck et al., 2001; Brinjikji et al., 2016; Bueno et al., 2021). Although some studies have reported associations between periodontal disease and carotid atherosclerosis, findings remain inconsistent due to differences in study design and residual confounding factors such as smoking (Duivenvoorden et al., 2013; Bonati et al., 2022; Wang et al., 2025). Carotid plaque burden is associated with increased systemic inflammation and oxidative stress, while elevated levels of inflammatory mediators, including hsCRP and IL-6, have been linked to plaque progression and instability (Howell et al., 2001; Hujoel et al., 2001; Zhang et al., 2026). Elevated hsCRP levels have also been reported in patients with concomitant periodontitis and carotid atherosclerosis (Desvarieux et al., 2003; Schillinger et al., 2006; Debing et al., 2008; Duivenvoorden et al., 2013).There is evidence suggesting that MMP-8 gene polymorphisms are associated with an increased risk of carotid atherosclerosis (Djurić et al., 2011; Mendoza et al., 2025; Xu et al., 2025).

Baseline Lp-PLA2 concentrations were not significantly correlated with measures of periodontal disease severity, suggesting that this biomarker may reflect systemic vascular inflammation and metabolic status rather than the extent of local periodontal tissue destruction. Similarly, MMP-8 and MPO showed only weak and non-significant associations with clinical periodontal parameters. Within this small exploratory pilot cohort, lower circulating levels of MMP-8 and Lp-PLA2 were observed following non-surgical periodontal therapy, whereas MPO and sCD40L exhibited non-significant downward trends. Given the absence of a control group, these findings should be interpreted as post-treatment associations rather than evidence of a causal effect of periodontal therapy on systemic inflammatory biomarkers. The unexpected inverse correlation between baseline sCD40L levels and PPD should not be considered conclusive. Given the small sample size, this finding may reflect statistical instability, the influence of individual extreme values, or residual confounding rather than a true inverse biological relationship. Therefore, the observed association should be considered exploratory and requires confirmation in larger, adequately powered studies.

The lower circulating MMP-8 and Lp-PLA2 levels observed following periodontal therapy may be consistent with a reduction in systemic inflammatory burden in this exploratory cohort. MMP-8 is involved in collagen degradation and extracellular matrix remodeling and has been implicated in plaque destabilization and vulnerability in atherosclerotic lesions (Herman et al., 2001). Similarly, Lp-PLA2 has been associated with vascular inflammation, lipid metabolism, and plaque activity, suggesting that lower post-treatment levels may reflect changes in systemic inflammatory pathways rather than local periodontal status alone (Duivenvoorden et al., 2013; Li et al., 2026). In contrast, MPO and sCD40L are influenced by multiple biological processes, including oxidative stress, platelet activation, and metabolic factors, which may partly explain the absence of statistically significant changes despite a numerical decrease observed after treatment (Li et al., 2026; Li et al., 2026).

Several limitations of the present study should be acknowledged. First, the study included a small sample size and no formal *a priori* sample size calculation was performed, as the investigation was designed as an exploratory pilot study. Recruitment was limited by the difficulty of identifying patients who simultaneously exhibited clinically relevant periodontitis, retained natural dentition suitable for periodontal assessment, and imaging-confirmed carotid plaque burden. Many patients with advanced imaging-confirmed carotid atherosclerotic plaques were elderly and presented with complete edentulism or implant-supported prosthetic rehabilitations, which reduced eligibility according to the study criteria. Consequently, the final cohort was small, and the results should be interpreted as exploratory and hypothesis-generating. Another limitation is the absence of post-treatment vascular imaging. Although carotid ultrasound was used to confirm carotid plaque burden at baseline, no follow-up imaging was performed; therefore, the present study cannot determine whether periodontal therapy was associated with changes in carotid plaque burden or plaque morphology. Finally, plaque index, gingival index, saliva, and gingival crevicular fluid biomarkers were not assessed, limiting the ability to directly link systemic biomarker changes with local periodontal inflammatory status.

If the results of this study are confirmed by further experimental research, periodontitis could represent another common and preventable factor contributing to the burden of carotid atherosclerosis, thereby providing clinicians and public health professionals with an additional opportunity to help reduce the impact of cardiovascular atherosclerotic diseases.

## 5 Conclusions

Inflammatory biomarkers represent important pathophysiological links between periodontitis and carotid plaque burden. This exploratory pilot study evaluated circulating inflammatory biomarkers in patients presenting with concomitant periodontitis and imaging-confirmed carotid plaque burden. Non-surgical periodontal therapy was associated with reductions in selected systemic inflammatory mediators, particularly MMP-8 and Lp-PLA2, whereas other biomarkers demonstrated non-significant changes.

Given the small sample size, absence of a control group, and exploratory study design, these findings should be interpreted cautiously and cannot establish causal relationships between periodontal therapy and vascular inflammatory status. In addition, no direct conclusions can be drawn regarding the association between periodontal disease severity and carotid plaque burden.

Nevertheless, the present results support the feasibility of further investigating inflammatory pathways potentially shared by periodontal and vascular disease in larger controlled prospective studies.

## Data Availability

The original contributions presented in the study are included in the article/supplementary material. Further inquiries can be directed to the corresponding author.
